# Designing Microflowreactors for Photocatalysis Using Sonochemistry: A Systematic Review Article

**DOI:** 10.3390/molecules24183315

**Published:** 2019-09-12

**Authors:** Swaraj Rashmi Pradhan, Ramón Fernando Colmenares-Quintero, Juan Carlos Colmenares Quintero

**Affiliations:** 1 Institute of Physical Chemistry, Polish Academy of Sciences, Kasprzaka 44/52, 01-224 Warsaw, Poland; 2 Universidad Cooperativa de Colombia, Calle 50A No. 41–34 Medellín, Colombia; ramon.colmenaresq@campusucc.edu.co

**Keywords:** ultrasound, flow microreactor, photocatalysis, water/air detoxification, organic synthesis, semiconductor

## Abstract

Use of sonication for designing and fabricating reactors, especially the deposition of catalysts inside a microreactor, is a modern approach. There are many reports that prove that a microreactor is a better setup compared with batch reactors for carrying out catalytic reactions. Microreactors have better energy efficiency, reaction rate, safety, a much finer degree of process control, better molecular diffusion, and heat-transfer properties compared with the conventional batch reactor. The use of microreactors for photocatalytic reactions is also being considered to be the appropriate reactor configuration because of its improved irradiation profile, better light penetration through the entire reactor depth, and higher spatial illumination homogeneity. Ultrasound has been used efficiently for the synthesis of materials, degradation of organic compounds, and fuel production, among other applications. The recent increase in energy demands, as well as the stringent environmental stress due to pollution, have resulted in the need to develop green chemistry-based processes to generate and remove contaminants in a more environmentally friendly and cost-effective manner. It is possible to carry out the synthesis and deposition of catalysts inside the reactor using the ultrasound-promoted method in the microfluidic system. In addition, the synergistic effect generated by photocatalysis and sonochemistry in a microreactor can be used for the production of different chemicals, which have high value in the pharmaceutical and chemical industries. The current review highlights the use of both photocatalysis and sonochemistry for developing microreactors and their applications.

## 1. Introduction

**Figure 1:**
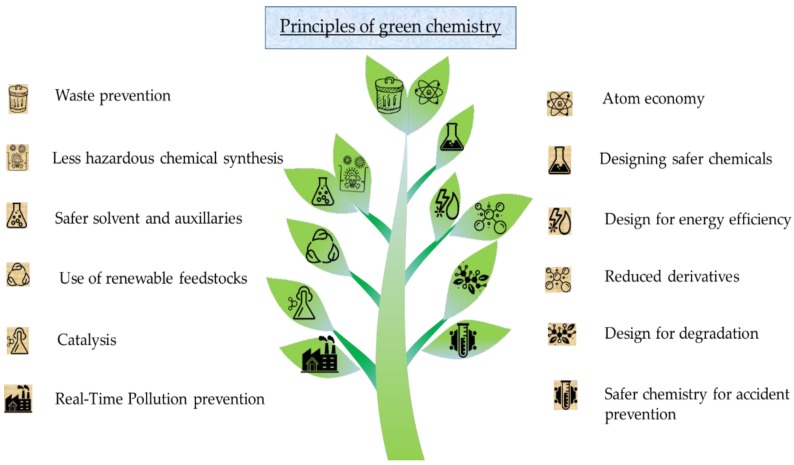
The twelve principles of green chemistry.

With the continuous and prosperous development of modern civilizations, environmental contamination has spread far and wide. Faced with this issue, humankind reached a consensus on the need for environmental treatment and remediation. Green chemistry is the implementation of twelve principles [1] (Figure 1) that lowers the use or generation of hazardous substances in the design, manufacture, and application of chemical products [2]. Our society is increasingly demanding the innovation of newer approaches to be sustainable in order to preserve the environment. It is crucial for these approaches to be less dependent on self-depleting sources or sources that effuse green-house gases in use.

Photocatalysis occupies an essential place in the ecological equilibrium and is a good example of green chemistry [3]. Photocatalysis activates reactions depending on the light (clean and superabundantly available from the Sun) as an energy source. Therefore, research on the utilization of solar energy has continued to be an important topic [2,4]. Photocatalysis in microreactors is attracting the attention of many researchers because of its greener aspect. Because of reduced reagent requirements, shorter reaction time, lessening of by-products, and minimized energy consumption, microreactors are regarded as a green synthetic approach [5]. Nowadays, many groups are working on synthesizing catalysts inside a microreactor. Among the catalysts, titania-based catalysts are well-known photocatalysts under UV (Ultraviolet) light and have been identified as a form of technology playing an important role in solving many of the problems in water purification [6]. 

In this review article, after a brief introduction, theoretical backgrounds of the flow microreactor, ultrasound, and their combined studies are discussed. Ultrasound irradiation is accepted as an environmentally benign technique to carry out chemical reactions [7]. The application of ultrasound waves has been considered as an agreeable technique in chemistry. Early works on catalyst synthesis in a microreactor using ultrasound and their comparison with conventional batch experiments and future challenges are reviewed in this article. 

**Figure 2:**
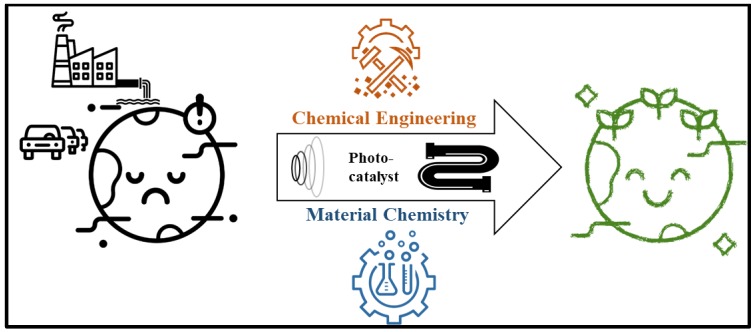
Two branches, trying to produce greener chemistry.

As illustrated in Figure 2, the scientific community is trying to make our planet green by combining chemical engineering (e.g., manufacturing microchannels by ultrasound) with material chemistry (e.g., photocatalysts). The major purpose of this review article is to highlight the challenges ahead of the design and development of (photo)catalytic microfluidic reactors using ultrasound. To the best of our knowledge, this is the first technical review in the field of microflow reactors for photocatalysis using sonochemistry, which is promising for the upcoming studies in this branch of science.

## 2. Theoretical Background

There are two main types of flow-reactor system that people use for synthetic photochemistry—micro and macro flow [8]. For various applications, people use the micro-synthesis technique in disciplines of both engineering and sciences. Definition of a microfluidic segment in a microreactor is described as a minimum unit having properties that can be used to improve various operations and reactions [5]. Microreactors offer new possibilities of reactions. Microreactors have been proven to be highly effective for catalytic reactions because of their indispensable advantages, such as uniform illumination without light attenuation, large surface-to-volume ratio and, consequently, attaining of a high heat and mass transfer rate, and the resultant satisfactory catalytic effect [9,10]. Also, one can easily control the contact time, shape, and size of the interface between fluids in these systems [11,12]. The aforementioned attributes make microreactors ideal for highly exothermic and fast reactions.

Recently, much attention has been paid to the development of microreactor technology for various applications, such as the synthesis of chemical compounds, environmental protection, biomedical and pharmaceutical studies, and healthcare, among others [9,13]. In a review article, Moraveji et al. discussed on two disadvantages of flow reactors—pressure drop and type of photocatalytic microreactor to be considered [14]. Yue et al. tried to overcome these issues by incorporation of a photocatalyst thin layer [10]. The potential development of more complicated flow reactions on progressively complex targets became viable because of the small volume of microreactors. Hence, the quantities of materials needed to optimize reaction conditions are greatly minimized, leading to reduce waste [15]. 

**Figure 3:**
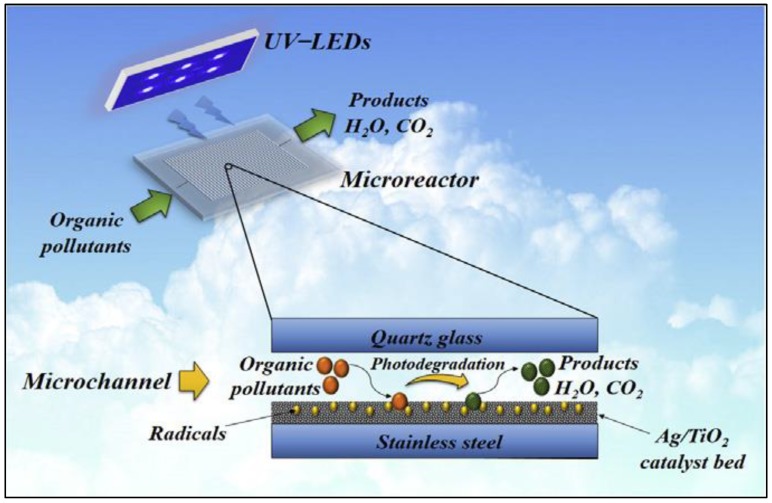
UV-LEDs-assisted preparation of silver-deposited TiO_2_ catalyst bed inside microchannels as a high-efficiency micro-photoreactor for cleaning polluted water. Reprinted from [18] with permission of Elsevier.

In the past decade, continuous flow microreactors have received considerable attention for performing organic transformations in safer and efficient ways. Even if microfluidic systems have a wide range of users in several fields, their commercialization is still limited [13]. It is now possible to reach the maximum selectivity of exothermic or endothermic, complex, extremely fast, and multiphase chemical reactions using a photocatalytic microreactor [16]. Very efficient degradation and different organic molecule synthesis, along with selective cleavage of peptides and proteins, have been done using micro-photoreactors immobilized with TiO_2_ catalyst, which can be very favorable for the synthesis of chemicals, pharmaceuticals, and proteomics [17]. Figure 3 shows a typical example of a photocatalytic microreactor used for wastewater treatment.

Ultrasound has been applied in several research fields. These include, but are not limited to, structural modification of materials, and their transmissions, imaging, medical treatment, materials processing, acoustic microscopy, and more recently, wireless communications [19]. Use of ultrasound in a liquid facilitates the breaking of chemical bonds through sonolysis process, resulting in the formation of free radicals. Acoustic cavitation, bubble formation in a liquid exposed to pressure fields, causes several chemical and physical effects [20]. These processes are useful for the synthesis of nanomaterials, incrementation of catalytic chemical reactions, destruction of pharmaceutical waste, wastewater treatment, degradation of organic pollutants, and are representative of a method of production of fuels [21]. In conventional systems, ultrasound is also used to intensify liquid-liquid processes because of its efficient agitation effects and non-invasive nature [19,22]. It accelerates chemical reactions by intensifying mass transfer. 

**Figure 4:**
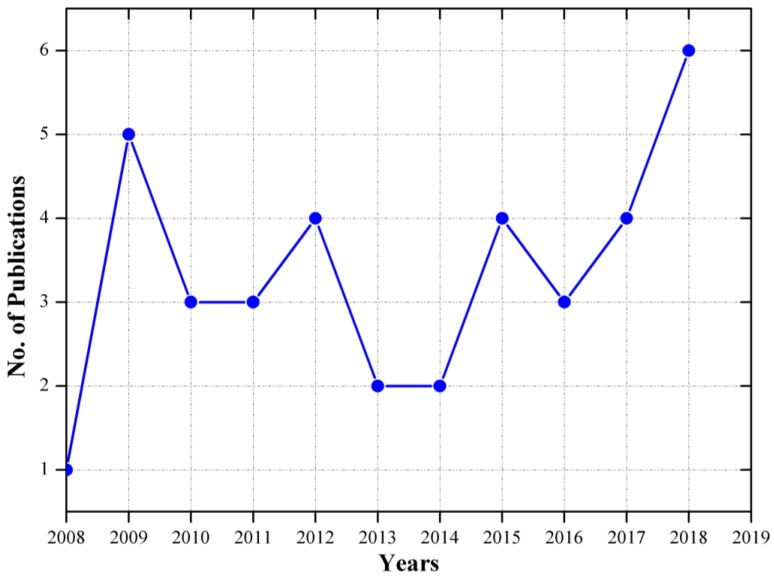
Work done with microreactors together with ultrasound in respective years (source: Scopus, access on 30 July 2019).

Currently, ultrasonics and microfluidics are introduced to revisit existing knowledge toolboxes to produce a technology-push hoping to commercialize modern inventions. Upon irradiation of an ultrasound wave, acoustic cavitation forms, such as cavitation microstreaming, shock wave, and jetting [23]. In this year (2019) until the time of submission, only five articles had been indexed by Scopus, which are retrievable by the combined keywords “microreactor” and “ultrasound.” The number of research articles published in the last decade is represented in Figure 4.

## 3. Side-by-Side Comparative Evaluation of Flow System to Batch

Microfluidics has many advantages compared with bulk chemistry, the first being slow diffusion. Therefore, to make the reaction faster, the distance required for interaction has to be smaller. The smaller channel dimensions also help to minimize the amount of sample required for analysis with reducing the by-products [13]. Recent advancement in this field has signified that miniaturization of reactors can be profitable in terms of kinetics, safety, and cost [24]. Because of its advantages, the synthesis of nanoparticles in microfluidics has become prominent in the past years [5]. The use of continuous microreactor led to the improvement of irradiation over the reaction mixture and offers a considerably reduced reaction time and better yields of products compared to batch reactors [25]. Batch reactors have a major disadvantage in the linear decrease in the intensity of the electromagnetic radiation with the square of the distance of the light source used [26,27]. The photocatalytic microreactors avoid this disadvantage by having a homogeneous illumination over the whole surface of the microchannel exposed to the light source [28]. Otherwise, the molecules undergoing photodegradation, under the control of the injection flow, constantly leave the reaction environment, avoiding the presence of by-products in the reaction mixture. The application of these devices to synthetic photochemistry started to spread from the 21st century [8]. 

There are several techniques for the prototyping of microfluidic systems [29], and different methods for the preparation of TiO_2_ films in photocatalytic microreactors [30]. These techniques should be fast and cost-effective from the design stage to the final system test. To fulfill the requirements, the production must be based on a simple technique and utilize low-cost instrumental resources [28]. Nanoparticle synthesis using continuous flow methods can produce a narrow size distribution of nanoparticles, which cannot be possible in a batch reactor. It has been proven that total reaction rate and photocatalyst mass transfer can be tuned with specific control, especially on size and shape, but also control over porosity, crystallinity, and thickness [14].

Noël et al. stated two important reasons for photochemistry achieving a remarkable increase in attention from researchers in academia and industry. The first reason is the exposure of visible light photo redox catalysis for organic synthetic chemistry. The second is the use of continuous-flow reactors [31]. In one of their publications, they compared their results in flow to those obtained in batch experiments [32]. They reported a negligible loss in activity when the reaction was performed in flow. In fact, in the flow reaction, they observed higher activity at very short residence times. This result concluded with the major advantages of flow chemistry. It stated that increased mass- and heat-transfer allows the flow reactor to have very fast and efficient heating. These properties make it ideal for fast reactions.

The same authors have also suggested a list of nine good reasons to utilize photo flow [31]. The reasons are as follows:

1. Improved irradiation of the reaction mixture;
2. Reliable scale-up;
3. Improved reaction selectivity and increased reproducibility;
4. Fast mixing;
5. Fast heat exchange;
6. Multiphase chemistry;
7. Multistep reaction sequences;
8. Immobilized catalysts;
9. Increased safety of operation.

Noël et al. developed a completely automated microfluidic system that can handle solids efficiently at high concentrations through acoustic irradiation [32]. They experimented with the amination reaction of aryl triflates, aryl bromides, and aryl chlorides. Working with the flow system assisted in carrying out the reactions at a very short time and in figuring out the conversions and yields accurately. They concluded that their system is ideal for multistep syntheses, which requires a heterogeneous reaction. Furthermore, microflow photocatalytic reactors have shown to be a competent setup compared to batch [33], as can be seen in the selective organic synthesis in heterogeneous photocatalysis in a microflow, which is still in an underdeveloped stage as compared to traditional batch systems.

## 4. Ultrasound: The Useful Tool for Chemists

**Figure 5:**
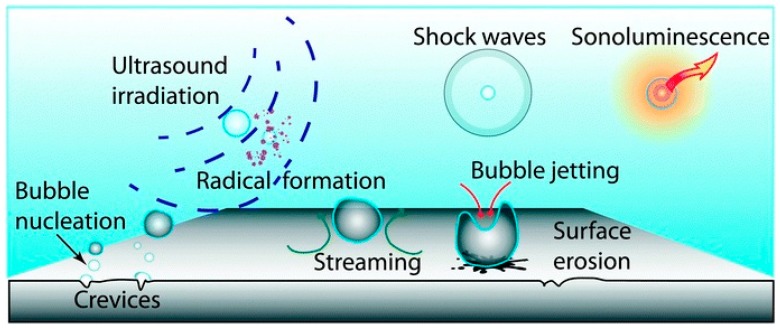
Effect of sonication. Reprinted from [20] with permission of the Royal Society of Chemistry.

Ultrasonic irradiation increases turbulence in the liquid phase, decreasing mass transfer limitations, and increasing the catalytically active surface area via the de-agglomeration and fragmentation of the particles [34]. Different effects of ultrasonic waves are shown in Figure 5. Nucleation, cavitation, bubble dynamics/interactions, thermodynamics, and chemical processes are the mechanisms of sonolysis.

Bridging and constriction are important mechanisms that lead to clogging in microfluidic devices, which can be eliminated via acoustic irradiation and fluid velocity, respectively [35]. Rivas et al., in their article, discussed the ultrasound approaches to control the particle formation inside the microchannel [33]. 

### 4.1. Synthesis of Materials

**Figure 6:**
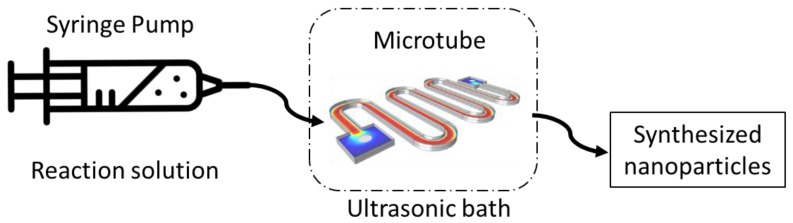
Diagram showing the use of ultrasound in a flow reactor for the synthesis of nanoparticles.

There has been a large amount of research in synthetic fields under ultrasonic environments, such as the synthesis of nano inorganic materials. There are comparatively fewer studies on the effect of solids on sonochemical activity [20]. Countless articles exist up until today on the use of ultrasound for material synthesis. This process deals with the formation, gradual growth, and bursting bubbles (Figure 5). Application of ultrasound to the solution, for nanomaterial synthesis, produces shock waves, leading to an increase in temperature and pressure necessary for chemical reactions [13] (a diagram has been given to demonstrate the synthesis of nanoparticles using microtube and ultrasonic bath, Figure 6). A simple, ultrasound-assisted wet impregnation method was applied to synthesize materials by Colmenares et al. [36]. 

### 4.2. For Immobilization of Catalyst 

Deposition of metal particles on a substrate by ultrasound is a process in which both the reduction of the oxidized metal precursor and the deposition of the resulting metallic particles are driven by ultrasonic irradiation. This technique has been employed to coat metallic particles on various substrates. Earlier investigations indicated that the technique could yield well-dispersed metal nanoparticles tightly adhered to the surface of a substrate [37,38]. It can be stated that ultrasound plays a vital role in developing thin-film of well-dispersed nanoparticles. Many researchers have taken advantage of ultrasound to immobilize nanoparticles. Recently, Liu et al. worked on the deposition of metallic platinum nanoparticles on CdS for photocatalytic hydrogen evolution using ultrasound [39]. However, the development of a more adaptable system that is more synthetically feasible is needed [32].

### 4.3. For Photocatalytic Experiments

In a review article, Qui et al. discussed heterogeneous sonocatalysts for treatment of organic pollutants in aqueous phase [40]. They discussed briefly the development of sonocatalysts from the past to the present in accordance with the different types of catalytic mechanisms. Teh et al., in another review article, discussed the development and modification of titania-based photocatalysts for pollutant-degradation using ultrasound technology [41]. They also stated the key operating parameters of ultrasound, followed by its application in the synthesis of the photocatalyst. Colmenares et al. synthesized magnetically separable materials by following the improved wet impregnation method assisted by ultrasonic irradiation. They developed a simple method for the preparation of magnetically separable TiO_2_/maghemite-silica photo-active nanocomposites. The resulting nanomaterials were further tested for their photocatalytic activities in the liquid phase of selective oxidation of benzyl alcohol in both aqueous and organic phase [36]. The unusual reaction conditions (extremely high temperatures and pressures forming quickly in liquids because of acoustic cavitation phenomena) of the ultrasonic irradiation technique were key factors in achieving homogeneously impregnated materials with nano-sized particles, and in the formation of heterojunctions. The catalysts were found to be highly photocatalytically active. Yu et al. also worked on photocatalyst synthesis [42]. They synthesized three-dimensional and thermally stable mesoporous TiO_2_ with high photocatalytic activity by high-intensity ultrasound-induced agglomeration.

## 5. Early Works on Microreactors

Microfluidic technology can be used profitably for the synthesis of nanomaterials and their catalytic studies. Efficient heat and mass transport in the miniaturized reaction chambers of microfluidic chips impart greater control at the molecular level. The microfluidic pathway offers an edge over the normal batch processes in terms of laminar flow, short molecular diffusion distance, and effective mixing [13,14,31]. Previously, many groups concentrated their work on exploring microfluidic photocatalytic microreactors for environmental application. Das et al. wrote a review article focusing on the fabrication techniques and operating parameters for this type of microreactor [43]. 

**Figure 7:**
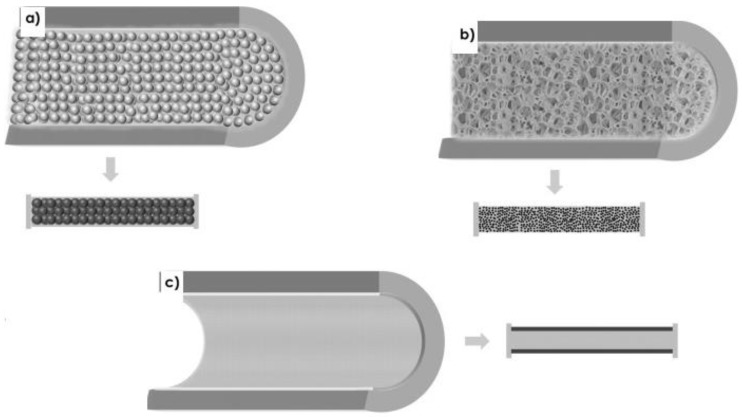
Schematic representation of the cross-section of a microchannel in (**a**) packed-bed, (**b**) monolithic, and (**c**) wall-coated microreactors. Reprinted from [9] with permission of Wiley.

Based on the method used to incorporate catalysts on the inner wall of the microreactor, it can be divided into three classes: (i) packed-bed, (ii) monolithic, and (iii) inner wall-functionalized [9] (Figure 7). The packed-bed reactor can be explained as the immobilization of a catalyst on insoluble support and is haphazardly assembled in the reactor, whereas in a monolithic reactor, the catalyst is made in the shape of structured material. In an inner wall-functionalized reactor, the catalyst is covalently attached to the interior wall of the reactor. To ensure a smooth flow of reagents, minimization in the mass transfer resistance was provided. Because of the complexity of the synthesis, their application is still limited [31]. Tao et al. proposed a synthesis procedure based on microfluidics for the production of Ag@Cu_2_O core-shell nanoparticles [44]. Sachdev et al. presented a microfluidic method for the synthesis of hollow Au shells and Fe_3_O_4_@Au core-shell nanoparticles within an emulsion droplet [45] (‘@’ stands for core-shell by the respective authors).

Flow chemistry has started to make an extensive impact on the way many chemists carry out synthesis over the last 15 years [8]. Microfluidics has significant applications in various fields [46,47]. In the year 2015, Yao et al. published a review article related to various applications of microreactors [5]. This review article was mainly based on structures and applications of microreactors in the synthesis of nanoparticles, and also on bio-substances, organics, and polymers. The whole article focused on multiphase microreactors. Knowles et al. used dual-channel microreactors for transformations, which are synthetically useful [8]. The same reactor was also applied for the synthesis of the antimalarial artemisinin, and the conversion of α-terpinene to ascaridole successfully. An additional application of microflow photochemistry includes the synthesis of vitamin D3 [7]. 

Nanoparticle synthesis in microreactor types for on-chip photocatalyst synthesis has been reviewed, along with challenges in handling the nanoparticles in microsystems [14]. The most important design parameter of photocatalytic reactors is the illuminated specific surface area of the photocatalyst. Matsushita et al. have developed a photocatalytic microreactor system, which has a considerably large surface area per unit volume [26]. Research over the past decade focused on enabling multi-step processes by developing complex microchemical systems. The prime example of such multistep microchemical synthesis is multi-step Heck synthesis carried out in continuous flow [19]. Microfluidic systems provide a platform for a broad range of syntheses. These allow automated optimization [48] and rapid experimentation (e.g., reaction conditions, catalysts) [49]. Moreover, microfluidic systems allow safe synthesis and increase the feasible reaction space (performing synthesis in supercritical solvents). Additionally, the residence time of species and the reactor temperature can be precisely controlled. All these studies focused on the use of microreactors for chemical synthesis in flows [19].

In a review article, the importance of continuous-flow photo-microreactors in water treatment, organic synthetic chemistry, and materials science was described [31]. Some recent examples pointed to complex applications, such as the synthesis of complex biologically active molecules [50]. Automated and self-optimizing flow processes have been developed to reduce manual labor [31]. In a recent article, Cambié et al. stated that a multidisciplinary approach would be the best strategy to overcome the remaining hurdles in chemistry. Intense collaborations between academia and industry are the most important part. To address the challenges of the future, industrial income has become more vital because of the drop in funding opportunities [31]. Considerable research in this field has been done in the last decade, and making further progress will be challenging. 

In another article, Shchukin et al. [51] stated the advantages of microflow photocatalytic process as (i) possibility of providing definite characteristics to the microreactor by removing additional functionalities; (ii) high active area for reaction with increased yield of photoreactions; (iii) less volume (micron and submicron), allowing one to perform photochemical synthesis in the highly organized solvent; (iv) reduced concentration and heating effects on adding reagents in the reaction; (v) possibility of modelling and mimicking photo-induced processes in nature on the micron and submicron level. So far, many articles have reported on several microns and submicron-confined environments for performing photocatalytic processes. However, there are only a few examples of spatially confined individual reactors for the semiconductor-catalyzed photodegradation reactions to date [49]. The study of reaction kinetics and mechanisms, influence of different parameters (e.g., size of the microreactor), adsorption of the reactants and intermediates, and solvent structure in the interior, among others, on the photosynthetic technique, along with a comparison of reaction products with those obtained by catalytic photolysis in non-confined media (e.g., in the slurry of dispersed photocatalyst) is scarce in the literature. These details can help in understanding the chemical and physicochemical processes occurring in the environment, as well as the development of spatially confined photosynthetic approaches. The results of conventional heterogeneous photocatalysis can be improved by exploiting the physical processes that occur in confined geometries with controlled diffusion of the reagents [51].

Various photocatalytic reactors have been reviewed for different applications [52,53]. Most of them can be classified into microreactors and slurry reactors. Some can be handled with suspended photocatalysts immobilized in the latter by considering the specific surface area of the catalyst and uniform light penetration in the reactor volume by various approaches [54]. The slurry reactors provide several active sites per unit volume. These microreactors were often used for air treatment [55]. Because of the limited designs available, photocatalytic reactors are still not commonly implemented in industrial processes. In the case of a three-phase microreactor with dispersing catalyst nano-powder, the higher adsorption rate was found in wastewater treatment. It has been seen that the photocatalytic activity decreases with particle size [56]. The mean particle size also can be easily adjusted by the pH of the solution and choice of solvent [57]. The accumulated particles inside a micro path make the recycling process difficult after the photocatalytic step [43]. 

**Figure 8:**
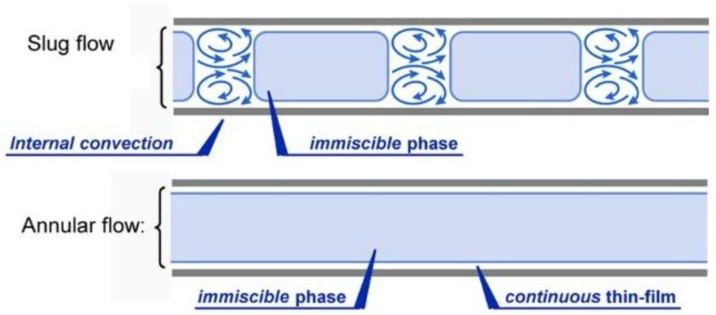
Particle formation in a single-channel microreactor. Reprinted from [31] with permission of American Chemical Society.

The typical flow systems found in the catalytic layer immobilized channel are slug flow or annular flow (Figure 8), depending on the operating conditions [58]. The important leverage of a microreactor with the immobilized thin-film catalyst is that it does not require a discrete step to separate the photocatalyst after the reaction. The high surface area of the catalyst also helps in increment of mass transfer in bulk and inter-phase. For example, oxygen that accepts electrons and, resultantly, does not allow recombination of electron-hole pair in the photocatalysis, leads to high reaction efficiency [59]. The lower interfacial catalyst surface area per mass is the main disadvantage of the inner surface-immobilized photocatalytic thin film of microreactors [60]. The combined effects of mass transfer with photocatalytic reaction have been studied in Charles et al.’s and Corbel et al.’s works [61,62].

Different microreactors have been developed to upsurge the reaction efficiency [63], such as micro-capillary reactors [64], single-microchannel reactors [18,65], and planar reactors, although it’s photocatalytic, as well as energy efficiency, still needs to be improved [66]. In a review article, Heggo et al. discussed the work of different researchers to attain high throughput. Some researchers tried to achieve this by increasing the length or number of microchannels, whereas others tried to enlarge the dimension by keeping one dimension in the microscale. 

In a review article, Woolley et al. discussed the materials (silicon, glass, and ceramics) and polymers (elastomers, thermoplastics, and paper) that scientists are using in microreactors for different purposes (microreactor’s fabrication). Hybrid devices have shown promising ability to gain the benefits of each material’s strengths [13]. Professor George Whitesides used polydimethylsiloxane (PDMS) to create inexpensive microfluidic devices, and Yoshida’s microreactor initiatives in Japan built up considerable interest in the microreactor area [5]. Das and Srivastava inspected various techniques to construct microstructures, such as mechanical micro-cutting, lithography, and etching technology. On the basis of the material of the devices, they divided the micro-photoreactors into four groups: ceramic microreactors, polymeric microreactors, metallic microreactors, and glass microreactors [43]. 

Signs of progress done in the modification and design of the structure of microreactors over the last ten year has been reported, and it has also introduced the improvement in organic reactions and synthesis of inorganic materials. Exemplary reviews have been published on the reaction process, the impact on downstream processing, and the product properties [67,68]. Multiphase microfluidic devices have also been discussed to synthesize inorganic and metal nanoparticles [5]. 

Some polymers, on the other hand, are presented as a good alternative for use in photochemistry and have been applied for the intensification of photochemical processes [69]. This paper focused on multichannel microreactors, which can be used for a wide range of liquid-phase organic synthesis reactions. The reactor system showed better potential because of the presence of several microchannels and the simplicity of parallelly arranging a number of these devices [69]. In a study, Ramos et al. investigated the possibility of employing UV-transparent polymer microtubes as supports for TiO_2_ (titanium dioxide) photocatalysts, and their applicability in the oxidation of organic pollutants [64].

Because of the advancement in syntheses such as increased mass and heat transfer, operational safety, the potential for purifying continuously, control over residence time, and scalability by parallel operation of several devices, the usage of microfluidic devices has attracted consideration from the pharmaceutical industry [70]. Despite these advantages, one of the biggest hurdles in the development of flow chemistry methods is the handling of solids, such as precipitates during the reaction, leading to clogging of the microchannels. Among all the approaches, the use of ultrasound is an effective way to avoid clogging. In order to prevent the particles from interacting with the reactor walls, segmented liquid-liquid flow can be used [71]. Though this is an efficient way to handle solids, the efficiency of the reaction can be reduced by using an additional solvent. Recently, Buchwald et al. presented a biphasic system of an organic solvent and water, which could solubilize both the organic and inorganic components of a reaction [72,73]. 

Microfluidic reactors have been developed to implement miniaturized laboratories for (i) synthesis of organic and inorganic compounds, and (ii) analytical tests and biomedical applications [28]. In these situations, the process parameters (P, T, V, and concentration) must be highly controlled in well-defined time units, in order to reduce raw material costs, analysis time, and risks in reagent handling, or potentially dangerous flammable, explosive, corrosive, and carcinogenic products, and bacteriological agents. From the advantages of the method, it can be stated that high temperature and long-time are not required. It is noteworthy that the diameter of the core-shell can be controlled by the concentration of the inner particle in the organic phase, and the diameter of hollow shells can be adjusted by varying the flow rate [14]. Y. Matsushita et al. examined the feasibility of the micro-reaction system on organic photoreactions, finding that the photocatalysis of TiO_2_ can be categorized into two types: homogeneous photocatalytic reaction and heterogeneous photocatalytic reaction systems. Among the different types of catalyst-based photochemical reactions, homogeneous-based photocatalysis has been broadly studied in microfluidic-based flow systems for selective organic synthesis [33]. 

## 6. Immobilization of Nanoparticles Inside the Microtube

**Figure 9:**
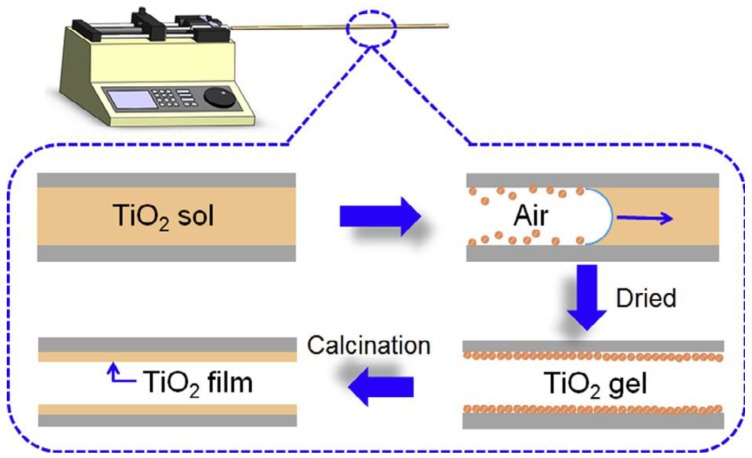
TiO_2_ thin film inside a microtube using the sol-gel method. Reprinted from [57] with permission of Elsevier.

Most research on photocatalytic reactions has been carried out using dispersed powders in conventional batch reactors. However, systems with the immobilized catalyst can avoid the separation of dispersed powders (preventing light penetration) after the reaction, as they have low interfacial surface areas. Thus, Matsushita et al. have developed photocatalytic microreactors with an immobilized TiO_2_ layer [74]. The thermal oxidation [75], physical vapor deposition (PVD) [76], chemical vapor deposition (CVD) [77,78], dip-coating [79], spin-coating [80], electrospun [81], sputtering [82], sol-gel [83], and electrodeposit [84] methods are techniques for the film formation step needed in the design of immobilized photoreactors. Figure 9 represents a sol-gel-based deposition of TiO_2_ inside a glass microtube.

Recently, Sohrabi et al. [14], in their review article, discussed the challenges as well as opportunities of microfluidic reactors. They stated that the main challenges in microfluidic nanoparticle synthesis and application are the crystallization of the photocatalyst, the poly-dispersity of particles and channel clogging, and the carryover of suspended photocatalysts. It would be worthwhile to devote much effort in the wall-coated microreactor by selecting suitable surfactants and manipulating polymerization conditions [85]. Lopez-Orozco et al. claimed that the high surface reactivity would enable the attachment of functional groups to synthesized microreactors inside nanocomposites or the microchannel. The evolution of the research on composite-based microreactors has been quite encouraging [86]. In the simplest case, the intrinsic activity of the wall of the reactor is sufficient to catalyze the reaction.

In most cases, a sufficient number of active sites cannot be provided by the surface of the microreactor or improve the existing surface area—some surface modification is required. Moreover, a surface pre-treatment can help to improve the adhesion of coatings to attain maximum potential for immobilization of the catalyst. Plasma oxidative treatment, thermal or chemical oxidation, UV radiation, anodic oxidation, and chemical modification are some methods that have been used for pre-treatment [87].

A microreactor with the photocatalytic thin film deposited on its inner spaces is a substitute for the slurry photocatalytic reactors [88]. H. Nakamura et al., in their article, discussed the modification of the inner wall of a microreactor and coating it for photocatalytic and enzymatic reaction studies. They used self-arrangement of colloidal particles to modify the microreactor inner wall. They observed an increase in conversion rate as well as yield [89]. Yue demonstrated the process of synthesis in microflow by improving heat and mass transfer rates. He explained some applications of catalytic processes in microfluidic reactors, for instance, selective hydrogenation, aerobic oxidation of alcohols, and direct hydrogen peroxide synthesis. He also discussed the multiphase flow in wall-coated microreactors and gas-liquid flow patterns in packed-bed microreactors [10]. The study on the amine N-alkylation processes in a microreactor with immobilized TiO_2_ has also been discussed earlier [26]. More examples of immobilized titania inside various types of microreactors are presented in the following Table 1. 

**Figure 10:**
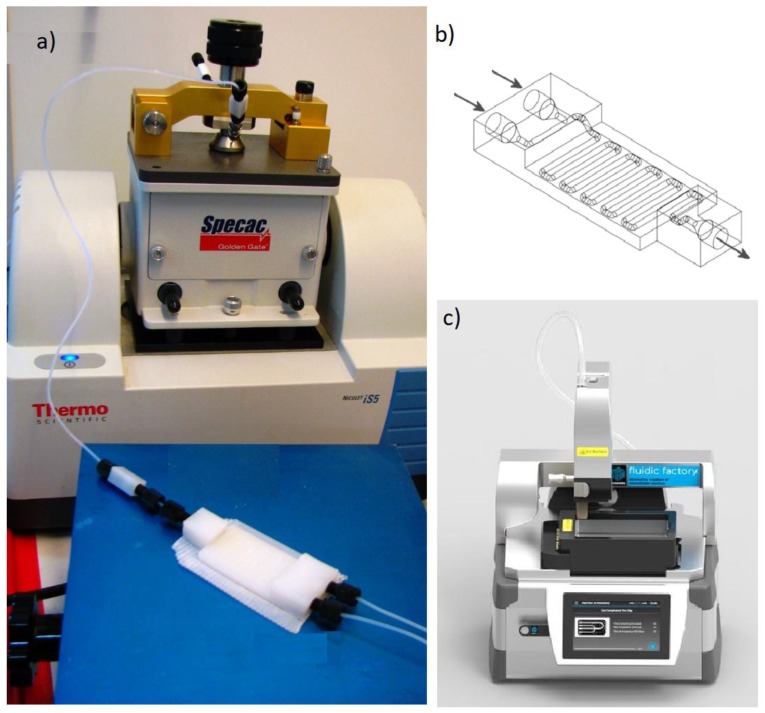
(**a**) Flow system setup and ATR-IR flow cell with connections. (**b**) Schematic representation of the three-dimensional (3D)-printed reactionware devices showing the internal channels (‘**a**’ and ‘**b**’ are reprinted from [95] with permission of American Chemical Society). (**c**) Picture of the Fluidic Factory 3D microdevice printer made by Dolomite Microfluidics.

On another note, many interesting novel contributions come from three-dimensional (3D) printed microchannels, which can be fabricated from plastic, metals, or glass. These types of microchannels can be made efficiently and quickly and are capable of manufacturing structures from microns to several centimeters. Different types of 3D printers are shown in the following Figure 10.

In a microfluidic device that was produced by rapid prototyping and was economically feasible and simple, a coating of TiO_2_ nanoparticles was applied, forming a photocatalytic microfluidic reactor destined to the degradation of organic dyes. It is important to point out that rapid prototyping of microfluidic devices is also relevant for the testing of small quantities of photocatalytic nanomaterials that are being developed in the research laboratories and that still lack the characterization of their photocatalytic efficiency. This new methodology will allow us to quickly test synthesized materials in reduced quantities, in addition to generating less waste. This more sustainable approach respects green chemistry requirements [96]. The possibility of varying the geometry of the microreactor, creating larger contact areas and a stronger bond between the photocatalytic coating and the surfaces of the microreactor, can further improve the photo-degradation efficiency of the microfluidic device, allowing for the increase of the flow velocity and, from this, the increasing of the volume of the treated solution. Different designs of the geometry of the device, implementation of a dye solution reflux system, photocatalyst chemical functionalization, and a light-emitting diode-based UV light system are being tested to improve the performance of the photocatalytic microreactor for potential applications in selective oxidation of functional groups of organic compounds [28].

Ultrasonic waves were used to break up agglomerations of particles [32,97]. The use of light transparent fluorinated ethylene propylene (FEP) microtubes (excellent visible light transmission, UV transmission: ~80%, temperature: −270 to 205 °C) with TiO_2_ leads to maximum usage of light for activating the photocatalyst for higher phenol degradation [93]. The design of a highly effective photoreactor is decisive to get the highest reaction rates with the immobilized form of a catalyst. Use of sonication for designing reactors, especially the deposition of a catalyst inside a microreactor, is a novel approach.

## 7. Photocatalytic Experiment 

The amount of light absorption of a photocatalyst at a given wavelength can be determined by the light intensity [98]. The photocatalyst activation step, the formation rate of electron-hole, is strongly dependent on the light intensity, and light distribution within the reactor undoubtedly determines the overall efficiency of the photocatalytic process. A light source of minimal space and lower photon cost is suitable for the microreactor system to take advantage of the miniaturized reaction vessel. Thus, Matsushita et al. employed UV-LEDs for the excitation light source of a photocatalyst [26]. Furthermore, it limited the depth of light penetration because of the absorption and scattering [99], as expressed by the Bouguer–Lambert–Beer law [100]. It should be noted that safety issues should be paid attention to, even in photochemical reactors for bio-applications [101], and with toxic or hazardous compounds [102]. Saien and Soleymani [54] explained the slurry photocatalytic microreactor as a favorable technique in dispersing TiO_2_ particles. Some experiments for the degradation of phenol used a high energy 125 W UV mercury lamp [52]. The manufacture of a microfluidic device with the nanostructured TiO_2_ coating has been described as being integrated on the inner surface of the microchannels in the work of Pandoli et al. Subsequently, efficiency was evaluated for the degradation of aqueous solutions of organic dyes in continuous flow under the action of UV light [28].

**Figure 11:**
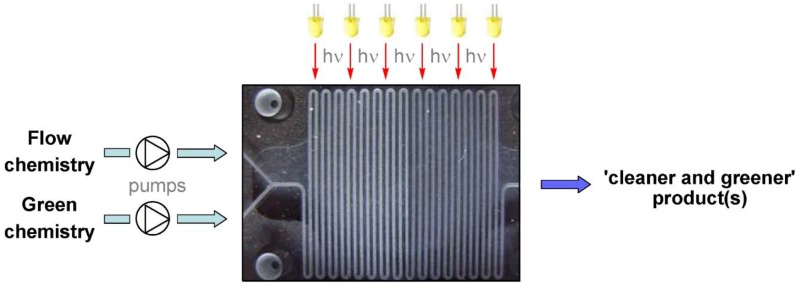
Concept of microflow photochemistry. Reprinted from [63] with permission of MDPI.

Currently, photochemistry using microspace is a major attraction of the scientific community for green chemistry application (Figure 11).

## 8. Microreactor with Ultrasound for Photocatalysis: A New Way Forward 

Sonochemical processes are highly efficient in terms of selectivity, reaction time, and operational simplicity [93,103,104,105,106] while being used for the synthesis of various semiconductor-based nanoparticles in batch reactions. Combination of the ultrasound transducer and the microfluidic reactor [107] has gained the attention of many researchers. Such designed systems can then be applied to microfluidic liquid-liquid extraction [108], degradation of contaminants [103,109,110], and particle synthesis [111,112]. These types of reactors are broadly used in laboratories and industrial applications, but the analysis and comparison of results obtained with them are extremely difficult, which has limited the scaling-up of sonochemical reactors in the industry [113].

**Figure 12:**
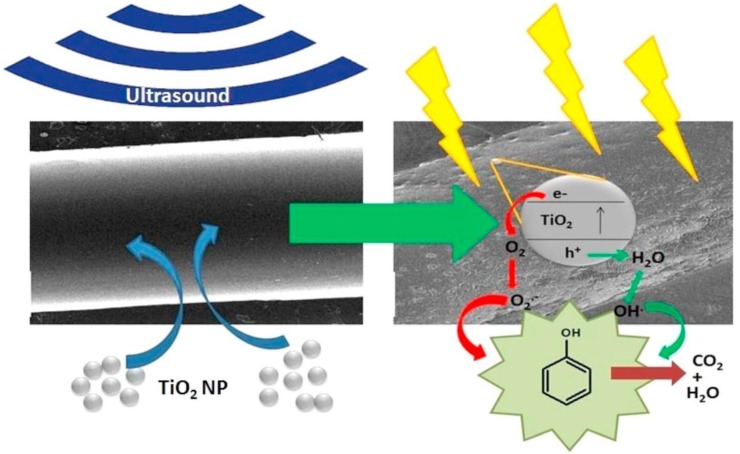
Photocatalytic phenol degradation in ultrasound (TiO_2_)-deposited FEP microtube. Reprinted from [93] with permission of Elsevier.

Many studies have been performed, and thus it is well verified that the advantages of ultrasound technique includes short reaction times, improved conversion, enhanced yields, and mild conditions [114]. A capillary microreactor, together with ultrasound, was designed and presented by Aljbour et al. to carry out some chemical reactions. They investigated the hydrolysis of benzyl chloride in a two-phase slug flow system. The increase in the rate of the hydrolysis reaction has been noticed with an increase in temperature, along with the effect of ultrasound. They noticed that the impact of ultrasound slowed down with an increase in the temperature. They also noted that the flow rate inside capillaries escalated the mass transfer between phases. The ultrasound helped in increment of the intensity of the internal circulations by splitting the large slugs into smaller sized slugs [7]. Sonication has been initially applied to homogeneous reactions; however, this approach has now been employed to heterogeneous reactions [115]. Ultrasound has some disadvantages, such as inefficient energy transfer via impedance and secondary effects such as streaming, sound field attenuation, heating, bulk mixing, emitter erosion, and sound emission. The parameters that influence sonochemical reactions and consider how they may be implemented to achieve systematic optimization has been discussed earlier [21]. Recently, Colmenares and co-workers were able to demonstrate for the first time an ultrasound-aided deposition of commercial TiO_2_ nanoparticles in an FEP-based microtube (Figure 12) using a probe-type ultrasonic system [93,94]. From AT-IR spectra, CH stretching peak in the modified tube, which was absent in the unmodified FEP microtube, confirmed that ultrasound brings some chemical changes in the inner walls of the FEP microtube. 

Current progress in photocatalysis on microreactor systems using ultrasound has been reviewed by Matsushita et al. They stated that the relative effect of ultrasound is more pronounced at a lower temperature compared to silent conditions. According to them, the reason is that the ultrasonic waves enhanced contact between reactants by damaging the phase boundary. In the silent condition, the contact between phases showed more mass transfer limitation. Increment of the vapor pressure of the liquid medium, as a result of elevated temperature, lead to easier effective cavitation [116]. This trend is more detectable at higher flow rates because of the lower exposure time to the ultrasonic irradiation. Rivas et al. focused on the control of cavitation as a means to improve the energy efficiency of sonochemical reactors, as well as in the solid handling with ultrasound. They discussed some examples of microfluidic clogging prevention, numbering-up, and scaling-up strategies. In their work, they tried to reduce the clogging of the microreactor and lengthen the operational time of the reactor [19]. Ultrasound-assisted capillary microreactors have also been proposed and tested as a potential reactor for the multiphase aqueous-organic system. The effect of ultrasound irradiation under different temperatures, capillary lengths, and flow velocity was also examined [7]. 

Sonochemistry could play a key role in overcoming limitations caused by solid formations by introducing ultrasound in conventional flow systems and microreactors [117,118]. Mass transfer limitation in microreactors can now be partially overcome by the help of ultrasound. The well-defined configuration of microreactors makes this easy and provides an ideal platform to investigate and control the acoustic cavitation process [118]. Colmenares et al. established a novel low energy (<80 °C) ultrasound-based deposition method using a probe-type ultrasonic system for coating of commercial TiO_2_ nanoparticles in the inner walls of FEP microtubes, knowing its importance in catalysis and photocatalysis fields [93,94]. The method is simple to implement and is environmentally friendly with low heat generation and has been filed for a patent [94]. The FEP microtube was pretreated with water using the ultrasound process, which resulted in physical changes of the inner surface of the FEP microtube, creating rough spots and an etched surface—facilitating the stable immobilization, under sonication, of TiO_2_ nanoparticles on FEP internal walls. It has been demonstrated that the change in the surface characteristics (functionalization by pretreatment and TiO_2_ nanoparticle deposition) of the inner walls of the fluoropolymer is due to the physical effect of ultrasound (a promising device for phenol degradation in water). Another work of Colmenares et al. reported, for the first time, the selective oxidation of benzyl alcohol to benzaldehyde in a photocatalytic microreactor under UV-LED as the light source [33]. In this work, they used an ultrasonic bath (with a temperature close to ambient) for immobilization of ZnO inside a microtube. 

## 9. Future Challenges and Conclusions

Since its introduction three decades ago, the field of microfluidics has witnessed significant growth in scientific research done across multiple disciplines, especially towards biological and medical applications. The advantages provided by the unique physical and chemical interactions of particles that take place inside the microscale channels, along with the coupling of multiple functionalities, has continued to drive the scientific advances of microfluidics. Outstanding research has been done in terms of materials and functions, their integration, and applications of microfluidics. In microfluidics, glass and silicon have been traditionally used most frequently, but recently polymeric materials have gained considerable attention, especially in the area of low-cost, and disposable devices. Still, there is a need to develop better material with improved properties, as the current generation of the material comes with its inherent advantages and disadvantages. The further improvement of the current method (e.g., different microreactor lengths, the application of different nanoparticles, physical and chemical effect optimization of ultrasound) will provide new ways, not only for environmental applications but also for new green organic synthesis protocols [93]. Even with this state of research, microfluidics has not been accepted outside of academia. However, acceptance of new technologies outside of academic research has always been slow, and more work should be done to promote wider and practical applicability of microfluidics. Heterogeneous photocatalysis in a microflow system for generation of value-added chemicals is a novel green chemistry approach requiring the understanding of photocatalysis, microfluidics, and reactor design. Research on the development of low energy and environmentally friendly-based photo-microreactor systems for photocatalysis is yet to be explored. In the areas of environmental and spatial analysis, effort should focus on creating robust and portable devices that can operate unattended for long periods. There are also some challenges related to 3D microreactors to be overcome, including chemical compatibility and operation at high pressures and temperatures [119].

The interesting use of ultrasound irradiation in catalyst synthesis is gaining more and more value from both the fundamental and application point of view. Sonication is giving us a great opportunity as a real green and cost-effective methodology and is foreseen to hold great potential in the near future [103]. Using low energy-based ultrasound for photocatalyst synthesis inside polymer-based microtubes (that does not deteriorate with age) will pave a new path towards the greener approach.

## Figures and Tables

**Table 1:** Immobilization of a catalyst inside different types of microreactors.

Reference	Type of Microreactor	Method of TiO_2_ Immobilization	Outcomes from TiO_2_ Characterization
[17]	metal-titanium foil	Anodization and hydrothermal treatment	Good mechanical properties of titania nanotube film, nanotubes of TiO_2_ (TEM, SEM)
[57]	glass capillaries	Sol-gel	Homogenous dispersion, narrow particle size distribution (SEM, TEM)
[18]	stainless steel microreactor	Sol-gel	Uniform distribution of catalyst on surface, crystalline size is 32 nm, the reflectance spectrum of pure TiO_2_ is 393 nm (HRTEM, XRD, DRS)
[74]	self-adhesive fluorine resin (EFEP) channel and switched between two glass plates	Sputtering	Growth of anatase peaks (XRD)
[90]	Silica capillary	Wash coating and calcination	The thickness of the deposited layer 88 nm (Field Emission Gun-Scanning Electron Microscopy(FEG-SEM))
[91]	Dual-film optofluidic microreactor	Hydrothermally prepared nanorod growth on fluorine-doped tin oxide (FTO) glass	2.4 μm thick film of TiO_2_ nanorods inside glass tube (SEM)
[92]	coil-type photoelectrocatalytic microreactor	Anodization	25 nm thickness and 12 to 15 µm length of titania nanotubes (FESEM)
[93,94]	fluorinated ethylene propylene (FEP) microtube	Ultrasound-based deposition	Structural transformation of polymer tube with ultrasound, thickness of catalyst layer was 3– 6µm (confocal microscopy, SEM)
